# Optimal immobilization position for conservative treatment of proximal humerus fractures by fracture type: a biomechanical cadaveric study

**DOI:** 10.1038/s41598-024-64326-8

**Published:** 2024-06-12

**Authors:** Seokhwan Jin, Joon-Ryul Lim, Tae-Hwan Yoon, Yun-Rak Choi, Yong-Min Chun

**Affiliations:** Department of Orthopaedic Surgery, Arthroscopy and Joint Research Institute, Severance Hospital, Yonsei University College of Medicine, 50-1, Yonsei-Ro, Seodaemun-Gu, Seoul, 03722 Korea

**Keywords:** Musculoskeletal system, Bone

## Abstract

In conservative treatment for proximal humerus fractures (PHFs), the immobilization position of the affected arm should not be determined uniformly. The aim of this study is to investigate the optimal immobilization position for conservative treatment of different types of PHFs. We hypothesized that the optimal position minimizing the deforming force in PHFs depends on the fracture components involved. PHF models involving either the surgical neck (SN) or greater tuberosity (GT) were created using 12 fresh-frozen cadaveric shoulders. In the SN model, the deforming forces on the pectoralis major muscle were measured in full adduction by increasing external rotation. In the GT model, the deforming force of the supraspinatus muscle was measured in neutral rotation by decreasing abduction, and the deforming force of the infraspinatus muscle was measured in full adduction by increasing internal rotation, respectively. In the SN model, the deforming force of the pectoralis major muscle increased significantly with external rotation from full internal rotation to neutral rotation (*P* = 0.006), indicating that the arm should be placed in full internal rotation. In the GT model, the deforming force of the supraspinatus muscle increased significantly with adduction from 45° of abduction to full adduction (*P* = 0.006); the deforming force of the infraspinatus muscle increased significantly with internal rotation from neutral rotation to full internal rotation (*P* = 0.006). These findings should be considered when placing the arm in abduction and neutral rotation so as to minimize the deforming force by either the supra or infraspinatus muscle. In conservative treatment for PHFs, the affected arm should be placed in a position that minimizes the deforming force on the fracture components involved.

## Introduction

In the elderly, proximal humerus fractures (PHFs) are the third most common non-vertebral fracture^1,2^ ranging from 4 to 10%^2–9^. Fortunately, in about 80% to 90% of them, it is well known that conservative treatment with immobilization for 3–4 weeks yields satisfactory outcomes^6,10,11^.

**Figure 1:**
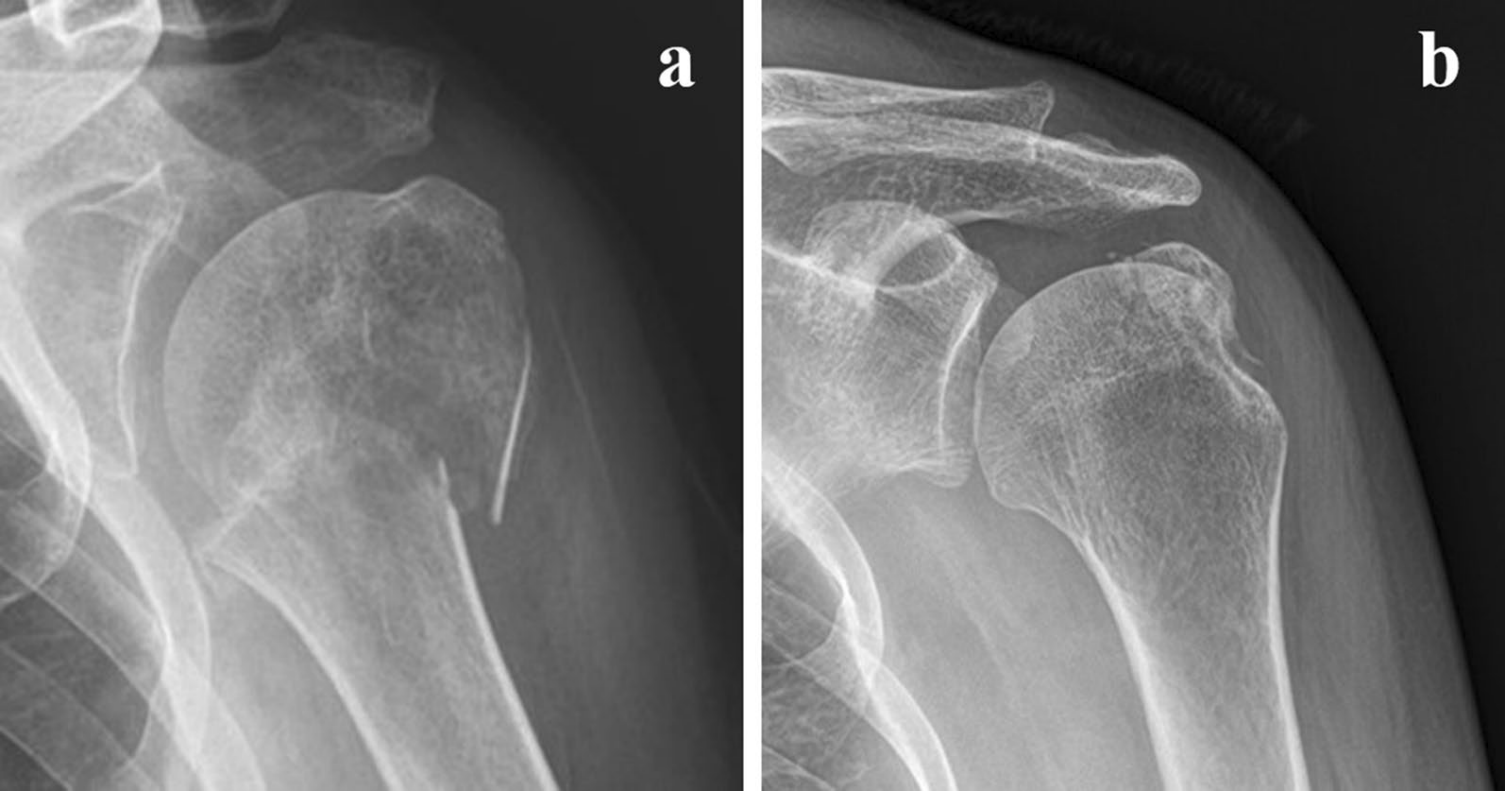
Reduction loss in proximal humerus fractures during immobilization. (**a**) Reduction loss of the surgical neck. (**b**) Reduction loss of the greater tuberosity.

PHFs mostly involve the surgical neck (SN) and/or greater tuberosity (GT) components^3,6^. In conservative treatment, it is important to immobilize the arm in a position that minimizes the deforming forces on the fracture components involved. Otherwise, no or minimal displacement may become worse and require surgical intervention during the immobilization period (Fig. 1a,b)^12,13^.

There has been little research on the immobilization position of the affected arm with consideration of fracture type. In the few studies touching on this topic, most of the focus was on varus collapse associated with SN fracture type^14,15^. There have been several cases where initial suboptimal immobilization positioning worsened the condition compared to the initial fracture^12,13^. In most cases, concomitant osteoporosis makes it challenging to achieve solid fixation even with surgical treatment after reduction loss compared to initial presentation.

The aim of this study was to investigate the optimal immobilization position of the affected arm for conservative treatment in different types of PHFs, taking into consideration the deforming forces acting on the fracture components. We hypothesized that in PHFs, the optimal position minimizing the deforming force would depend on the fracture components involved.

## Methods

### Specimen preparation and deforming force measurement

**Figure 2:**
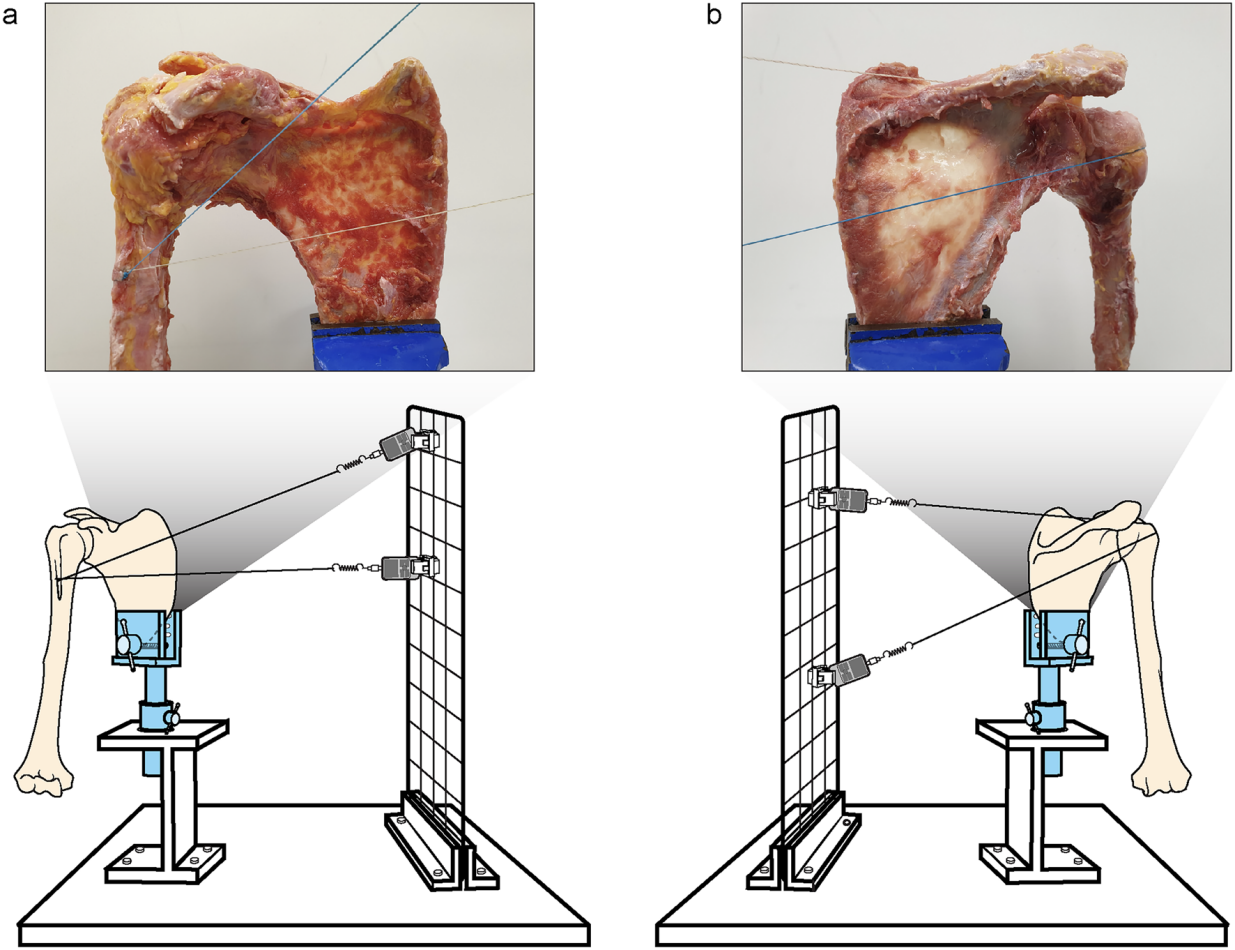
Schematic illustration of the custom jig setup. (**a**) In the surgical neck model, two FiberWires represent the two heads of the pectoralis major muscle. (**b**) In the greater tuberosity model, two FiberWires represent the supraspinatus and infraspinatus muscles. All FiberWires were connected to the digital force gauges along their respective anatomic force vectors).

In this study, 12 fresh-frozen cadaveric shoulders were used; the age of the cadavers ranged from 65 to 88 years (mean age, 79 ± 8 years). There were four male and eight female cadavers. None of the specimens had a history of trauma or surgery and appeared normal upon visual inspection. They were frozen and stored at -20℃ and were defrosted overnight at room temperature prior to use. The skin, soft tissues, and muscles around the shoulder (scapula, clavicle, and humerus) were dissected except the rotator cuff muscles and the pectoralis major muscle, carefully preserving the capsular structures without any damage. The humerus and scapula remained intact. After completing specimen preparation, the scapula was fixed to a custom jig (Fig. 2) using a scapular clamp with a 20° anterior tilt in the sagittal plane^16–18^.

In the SN model (PHFs involving the SN), the clavicular and sternocostal heads of the pectoralis major muscle may act as the deforming force responsible for displacement by pulling the shaft anterior and medially^6,19–21^. In the GT model (PHFs involving GT), the supraspinatus and infraspinatus muscles may act as the deforming force responsible for displacement by pulling the GT either superiorly or posteriorly^6,19–21^. Our design measured the tension applied on the fracture components and how it changed depending on arm position in order to investigate the optimal arm position for minimizing deforming force on the fracture components.

The direction of the suture (FiberWire; Arthrex, Naples, FL, USA) was determined along the anatomic force vectors of the representative muscles or tendons^18,22^, taking into account their centers of origin and insertion (Fig. 2). In the SN model, considering that the pectoralis major muscle has both clavicular and sternocostal heads, it was replaced by two sutures. In the GT model, the supraspinatus and infraspinatus muscles were each replaced by a single suture. Based on the footprint of each muscle or tendon on the humerus, sutures replacing each head of the pectoralis major muscle were also transosseously secured by creating holes in the center of the lateral lip of the bicipital groove. The suture replacing either the supraspinatus or infraspinatus muscle was secured transosseously through holes in the center of the superior and middle facets of the GT, respectively. Each suture was attached to the end of a spring (Sciencelove, Goyang, Korea) with a constant elastic modulus(*k*) of 0.417 N/mm that was connected to a digital force gauge (AMF-30; Aliyiqi, Zhejiang, China), allowing free movement and fixation in the sagittal plane. Then, the starting point was set after pre-tensioning to 0.5 N^23^.

For each fracture model, before the changes in tension were measured by increasing the deforming force according to arm position, the reference zero-point for each fracture was determined, considering the various types of shoulder immobilization braces available on the market (a sling in internal rotation, an abduction brace and a brace in neutral rotation). For the reference point of the SN model where the pectoralis major may cause medial displacement of the humerus, full adduction (0° of abduction) and full (90°) internal rotation of the arm were determined where the tension loaded by the two heads of the pectoralis major muscle was minimized. Then, tension was measured in full adduction by gradually increasing external rotation from 90° of internal rotation to 0° (neutral rotation) in decrements of 30°. For the GT model, although both the supraspinatus and infraspinatus muscles insert through the GT, tension loaded on each tendon was measured separately because the directions of the vectors were different. As the reference point for where the tension is loaded by the supraspinatus (abductor inserting on the GT), 45° of abduction along the scapular plane^16,18^ and internal rotation of 30° from the coronal plane^18,24^ was used. Tension was measured during adduction in decrements of 15°. Finally, as the reference point for where the tension is loaded by the infraspinatus (external rotator insertion on the GT), full adduction and neutral rotation (0° of rotation) of the arm were determined. Then, tension was measured while gradually increasing internal rotation from 0° (neutral rotation) to 90° of internal rotation in full adduction in decrements of 30°.

### Statistical analysis including sample size calculation

Since there has been no previous studies addressing a similar topic, the study design was determined after consulting medical statisticians. Then, a pilot study was conducted using three cadaveric specimens. The sample size was calculated using the difference in deforming force caused by the infraspinatus muscle when moving from 0° (neutral rotation) to 60° and from 0° (neutral rotation) to 90° of internal rotation. The mean ± standard deviation value for differences between each group in the pilot study was 1.3 ± 1.5 N. Based on these data, 12 specimens were needed to present 80% power at an α level of 0.05.

The Friedman test was used to identify significant differences among at least one group (an increase in deforming force due to arm movement) out of three for each muscle. The Wilcoxon signed-rank test was used for post hoc analysis for significant differences from the Friedman test with a Bonferroni correction. The Jonckheere-Terpstra test was used to determine either positive or negative trends in tension caused by each muscle across three groups of arm movement. The level of statistical significance for all tests was set at *P* < 0.05. All statistical analyses were conducted using SPSS Statistics for Windows (Version 27.0; IBM Corp., Armonk, NY, USA).

### Eithics statement

All cadavers used in this study were legally donated to the Surgical Anatomy Education Center, Yonsei University College of Medicine. Donors of cadavers approved the cadavers for use in research. The study was authorized by the Institutional Review Board of Yonsei University Health System, Severance Hospital (4–2023-0826). All experiments were performed in accordance with relevant guidelines and regulations.

## Results

In the SN model, at least one of three groups of arm movement showed a significant difference when compared to the others (all *P* < 0.001, Table 1). The increase in tension caused by the clavicular head of the pectoralis major muscle was 3.7 ± 1.0 N with external rotation from full internal rotation to neutral rotation was significantly greater (*P* = 0.006, Table 2) than other groups of arm movement (1.0 ± 0.4 N with external rotation from full internal rotation to 60° and 2.0 ± 0.7 N with external rotation from full internal rotation to 30°). Likewise, the increase in tension caused by the sternocostal head of the pectoralis major muscle was 4.6 ± 1.3 N with external rotation from full internal rotation to neutral rotation, which was significantly greater (*P* = 0.006, Table 2) than other groups of arm movement (1.4 ± 0.6 N and 2.7 ± 0.9 N). The more the arm was externally rotated, the more the tension increased (all *P* < 0.001, Table 3).

**Table 1:** Increase in tension according to the arm movement of each muscle either in the surgical neck or greater tuberosity model.

	Arm movement	Mean ± SD (N)	Overall *P*-value
Pectoralis major muscle, clavicular head	ER 0°–30°	1.0 ± 0.4	< 0.001
	ER 0°–60°	2.0 ± 0.7	
	ER 0°–90°	3.7 ± 1.0	
Pectoralis major muscle, sternocostal head	ER 0°–30°	1.4 ± 0.6	< 0.001
	ER 0°–60°	2.7 ± 0.9	
	ER 0°–90°	4.6 ± 1.3	
Supraspinatus muscle	Abd 45°–30°	0.5 ± 0.3	< 0.001
	Abd 45°–15°	1.2 ± 0.3	
	Abd 45°–0°	2.2 ± 0.4	
Infraspinatus muscle	IR 0°–30°	1.4 ± 0.5	< 0.001
	IR 0°–60°	2.5 ± 0.9	
	IR 0°–90°	3.8 ± 1.4	

The values are presented as mean and standard deviation. *ER* External rotation, *Abd* Abduction, *IR* Internal rotation.

**Table 2:** Post-hoc test of values within each muscle either in the surgical neck or greater tuberosity model.

	Arm movement	*P*-value
Pectoralis major muscle, clavicular head	1.0 ± 0.4 (ER 0°–30°) vs. 2.0 ± 0.7 (ER 0°–60°)	0.006
	2.0 ± 0.7 (ER 0°–60°) vs. 3.7 ± 1.0 (ER 0°–90°)	0.006
	3.7 ± 1.0 (ER 0°–90°) vs. 1.0 ± 0.4 (ER 0°–30°)	0.006
Pectoralis major muscle, sternocostal head	1.4 ± 0.6 (ER 0°–30°) vs. 2.7 ± 0.9 (ER 0°–60°)	0.006
	2.7 ± 0.9 (ER 0°–60°) vs. 4.6 ± 1.3 (ER 0°–90°)	0.006
	4.6 ± 1.3 (ER 0°–90°) vs. 1.4 ± 0.6 (ER 0°–30°)	0.006
Supraspinatus muscle	0.5 ± 0.3 (Abd 45°–30°) vs. 1.2 ± 0.3 (Abd 45°–15°)	0.006
	1.2 ± 0.3 (Abd 45°–15°) vs. 2.2 ± 0.4 (Abd 45°–0°)	0.006
	2.2 ± 0.4 (Abd 45°–0°) vs. 0.5 ± 0.3 (Abd 45°–30°)	0.006
Infraspinatus muscle	1.4 ± 0.5 (IR 0°–30°) vs. 2.5 ± 0.9 (IR 0°–60°)	0.006
	2.5 ± 0.9 (IR 0°–60°) vs. 3.8 ± 1.4 (IR 0°–90°)	0.006
	3.8 ± 1.4 (IR 0°–90°) vs. 1.4 ± 0.5 (IR 0°–30°)	0.006

The values are presented as mean and standard deviation. *ER* External rotation, *Abd* Abduction, *IR* Internal rotation.

**Table 3:** Trend analysis of each muscle according to arm movement either in the surgical neck or greater tuberosity model.

	Arm movement	Mean ± SD (N)	Standard J-T Statistic	Trend *P*-value
Pectoralis major muscle, clavicular head	ER 0°–30°	1.0 ± 0.4	5.252	< 0.001
	ER 0°–60°	2.0 ± 0.7		
	ER 0°–90°	3.7 ± 1.0		
Pectoralis major muscle, sternocostal head	ER 0°–30°	1.4 ± 0.6	5.295	< 0.001
	ER 0°–60°	2.7 ± 0.9		
	ER 0°–90°	4.6 ± 1.3		
Supraspinatus muscle	Abd 45°–30°	0.5 ± 0.3	6.006	< 0.001
	Abd 45°–15°	1.2 ± 0.3		
	Abd 45°–0°	2.2 ± 0.4		
Infraspinatus muscle	IR 0°–30°	1.4 ± 0.5	4.990	< 0.001
	IR 0°–60°	2.5 ± 0.9		
	IR 0°–90°	3.8 ± 1.4		

The values are presented as mean and standard deviation. *ER* External rotation, *Abd* Abduction, *IR* Internal rotation.

In the GT model, at least one of three groups of arm movement showed a significant difference when compared to the others (all *P* < 0.001, Table 1). The increase in tension caused by the supraspinatus muscle was 2.2 ± 0.4 N with adduction from 45° of abduction to full adduction, creating significantly greater tension (*P* = 0.006, Table 2) than other groups of arm movement (0.5 ± 0.3 N and 1.2 ± 0.3 N). The more the arm was adducted, the more the tension increased (*P* < 0.001, Table 3). The increase in tension caused by the infraspinatus muscle was 3.8 ± 1.4 N with internal rotation from neutral rotation to full internal rotation, which was significantly greater (*P* = 0.006, Table 2) than other groups of arm movement (1.4 ± 0.5 N and 2.5 ± 0.9 N). The more the arm was internally rotated, the more the tension increased (*P* < 0.001, Table 3).

## Discussion

In this study, we measured the tension exerted by muscles on each fracture model to determine the optimal immobilization position of the affected arm for conservative treatment in different types of PHFs. In the SN model, the position minimizing tension by the pectoralis major was full internal rotation in full adduction. In the GT model, while the tension exerted by the supraspinatus muscle was minimized in 45° of abduction in 30° of internal rotation, the tension exerted by the infraspinatus muscle was minimized in neutral rotation in full adduction.

At our tertiary hospital, we frequently encounter cases of failed conservative treatment for PHFs due to suboptimal immobilization position. Upon reviewing previous studies^14,15,19,25,26^, many kinds of slings or braces that achieve internal rotation of the shoulder have been conventionally utilized for conservative treatment of PHFs. In recent studies^20,21,27^, however, there has been a proposal to immobilize the arm in the neutral position in PHFs. On the other hand, Chalmers et al.^14,15^ suggested shoulder abduction and internal rotation for the immobilization position in surgical neck fractures to decrease deformation by muscular force. As described in previous studies, there is still no consensus on optimal immobilization position for conservative treatment in different types of PHFs. Before applying an immobilization brace, the anatomy of the fracture component and subsequent deforming muscular forces need to be fully understood. In addition, the optimal position for immobilization should not be determined uniformly, but after considering every circumstance such as comminution and instability of the involved fracture components, among other factors. Even in the GT model, deforming muscular forces of the supraspinatus and infraspinatus muscles may vary, and the optimal position should be identified through research. If the SN and GT are involved at the same time, the optimal immobilization position should be chosen to minimize displacement of the more unstable fracture component, and this should be monitored closely.

**Figure 3:**
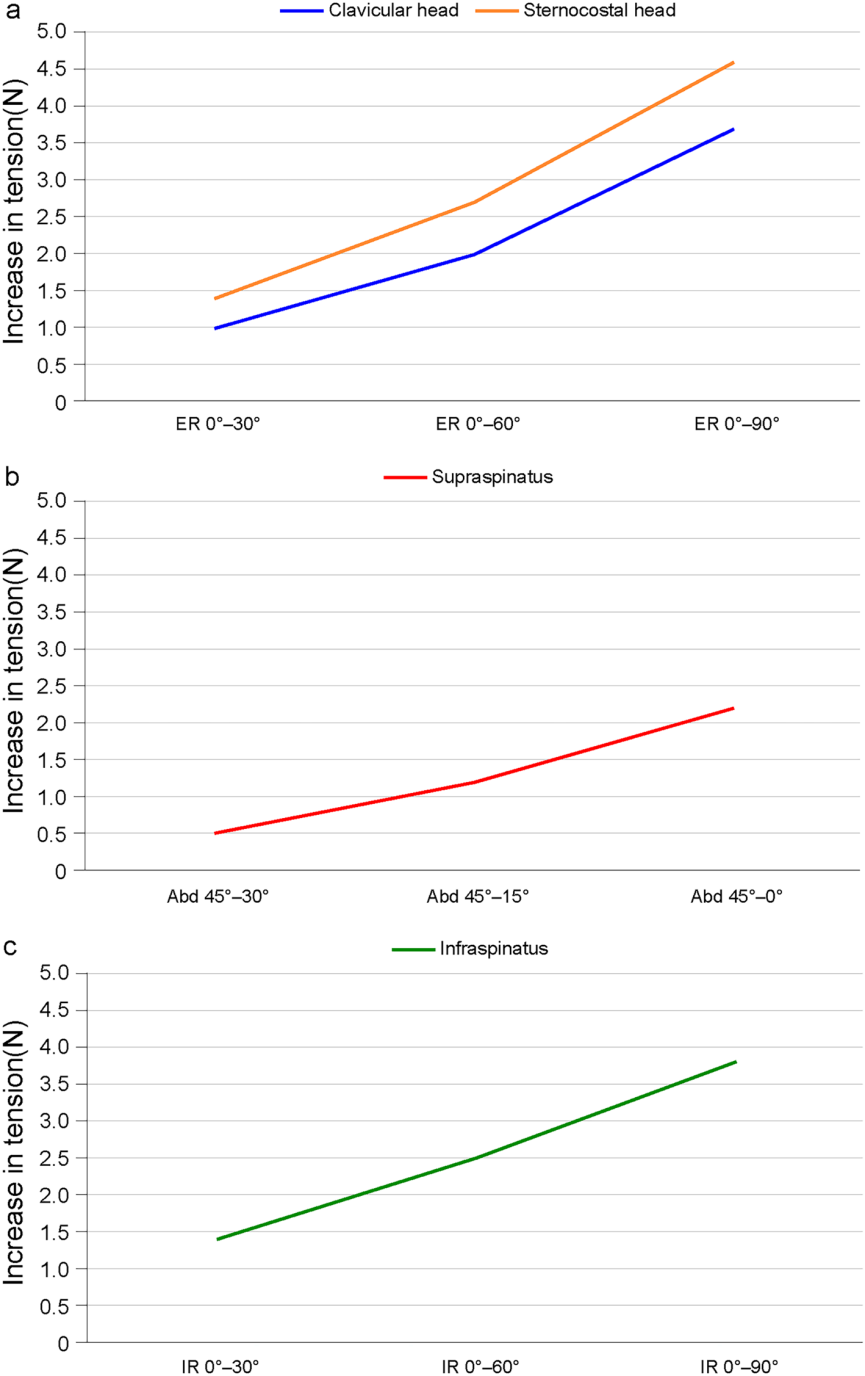
Increase in tension according to arm movement. (**a**) Two heads of pectoralis major muscle, (**b**) supraspinatus muscle, (**c**) infraspinatus muscle. *ER* External rotation, *Abd* Abduction, *IR* Internal rotation.

The current study found that in the SN model, it is necessary to immobilize the arm such that the deforming force exerted by the pectoralis major muscle, which pulls the shaft component anteriorly and medially, is minimized^6,19–21^. As described in our results, the increase in tension in the SN fracture model was greatest from full internal rotation to neutral rotation (Fig. 3). Thus, to minimize the deforming force of the pectoralis major, developing medial displacement of the shaft component in the SN fracture model, the affected arm would be better immobilized in adduction and full internal rotation. In contrast, in the GT fracture model, immobilization in a sling, where the affected arm is placed in adduction and full internal rotation, posterior displacement of the GT can develop. In the GT model, the tension exerted by either the supraspinatus or infraspinatus muscle varied and depended on arm position (Fig. 3); while we need to be cautious in interpreting and generalizing these results, the arm would be better immobilized in 45° abduction rather than full adduction in terms of the supraspinatus, and in neutral rotation rather than other internal rotations in terms of the infraspinatus to minimize the deforming force on each tendon. In cases of a combination of SN and GT fractures, the optimal position will be dependent on the severity of comminution and displacement of each component. Thus, the optimal position may need to be identified through several trials of various immobilization braces.

This study has several limitations. First, we did not address the varus failure caused by medial calcar comminution, which is frequently encountered during conservative treatment of surgical neck fractures. This was because it was difficult to simulate the extent of medical calcar comminution through a cadaver study. Second, the spring used in the study had a constant elastic modulus (0.417 N/mm) regardless of the change in length. In real muscles, the elastic modulus varies as the length changes. Therefore, the trends in observed increases or decreases in deforming forces based on arm position are more relevant than the absolute values of deforming force obtained using the spring. Third, each muscle was replaced by one or two FiberWires along the anatomic force vectors taking into consideration its origin and insertion. However, real muscles are voluminous structures extending from origin to insertion. The direction of the forces exerted by actual muscles on fracture components may differ from the direction of forces applied by the FiberWire.

## Conclusion

In conclusion, in conservative treatment for PHFs, the affected arm should be immobilized in the position that minimizes the deforming forces of relevant tendons or muscles on the fracture components.

## Data Availability

The datasets analyzed during the current study are available from the corresponding author on reasonable request.
